# Determining and addressing obstacles to the effective use of long-lasting insecticide-impregnated nets in rural Tanzania

**DOI:** 10.1186/1475-2875-8-315

**Published:** 2009-12-31

**Authors:** Maria Widmar, Courtney J Nagel, Deborah Y Ho, Peter W Benziger, Nils Hennig

**Affiliations:** 1 Mount Sinai School of Medicine, One Gustave L Levy Place, New York, NY 10029, USA; 2 Tufts University School of Medicine, 136 Harrison Avenue, Boston, MA 02111, USA; 3 Global Health Center, Mount Sinai School of Medicine, One Gustave L Levy Place, Box 1255, New York, NY 10029, USA

## Abstract

**Background:**

The objective of this project was to achieve high, sustainable levels of net coverage in a village in rural Tanzania by combining free distribution of long-lasting insecticide-impregnated nets (LLINs) with community-tailored education. In Tanzania, malaria is the leading cause of morbidity and mortality. Although malaria bed nets have a well-established role in reducing disease burden, few rural households have access to nets, and effective use depends on personal practices and attitudes.

**Methods:**

Five practices and attitudes inconsistent with effective LLIN use were identified from household interviews (n = 10). A randomized survey of villagers (n = 132) verified local prevalence of these practices and attitudes. Community leaders held an educational session for two members of every household addressing these practice and attitudes, demonstrating proper LLIN use, and emphasizing behaviour modification. Attendees received one or two LLINs per household. Surveys distributed three weeks (n = 104) and 15 months (n = 104) post-intervention assessed corrected practices and attitudes. Project efficacy was defined by correction of baseline practices and attitudes as well as high rates of reported daily net use, with statistical significance determined by chi-square test.

**Results:**

Baseline interviews and surveys revealed incorrect practices and attitudes regarding 1) use of nets in dry season, 2) need to retreat LLINs, 3) children napping under nets, 4) need to repair nets, and 5) net procurement as a priority, with 53- 88.6% incorrect responses (11.4-47% correct responses). A three-week follow-up demonstrated 83-95% correct responses. Fifteen-month follow-up showed statistically significant (p < 0.01) corrections from baseline in all five practice and attitudes (39.4-93.3% correct answers). 89.4% of respondents reported using their nets every night, and 93.3% affirmed purchase of nets as a financial priority.

**Conclusions:**

Results suggest that addressing community-specific practices and attitudes prior to LLIN distribution promotes consistent and correct use, and helps change attitudes towards bed nets as a preventative health measure. Future LLIN distributions can learn from the paradigm established in this project.

## Background

In Tanzania, malaria is the leading cause of morbidity and mortality in both adults and children under five [1,2]. 97% of the population is currently considered at risk for infection, with 75% living in high intensity transmission areas [3]. This burden of disease costs Tanzania an estimated 3.5% of its GDP [2], significantly inhibits long-term growth and development, and translates into school absenteeism, low workplace productivity, and reduced agricultural production [4,5].

The rural Kagera region of Tanzania, the site of this project, ranks first nation-wide in malaria incidence [2]. Poor transportation and limited medical resources in Kagera make access to treatment challenging and often unfeasible. In this setting, preventative measures are essential in reducing malaria-related morbidity and mortality. The insecticide-treated net (ITN) is currently the most widely utilized and well-studied preventative tool, shown to provide 23% more protection than untreated nets [6]. In recent years, methods for optimizing ITN use have been described. For example, studies document a "mass effect" in which children not sleeping under a net but living within 0.6 to 1.5 km of areas with 80-96% net coverage were at decreased malaria risk [7-9]. While "mass effect" is attributable to the combined insecticidal power of all nets in a definable area, this power is reduced by the fact that more than 50% of ITN owners do not regularly retreat their nets after the advertised interval [8-10]. One successful approach to this problem is the use of World Health Organization (WHO) recommended long-lasting insecticide-impregnated nets (LLINs), found to be 95% effective in inhibiting blood feeding even after seven years of use [11].

Despite the increasing use of LLINs to sustain maximal insecticidal power, achieving the high coverage rates necessary for "mass effect" has been a challenge in Tanzania. Three manufacturing companies in Tanzania produce over four million nets per year for local use and export, and yet the average cost of an ITN is TSH 4500 (US$3.50), largely beyond the pecuniary means of rural subsistence farmers. Thus, there exists a significant discrepancy in net ownership between rural and urban areas. While 71% of urban households have net coverage, in some rural communities it is as low as 10% [12]. LLINs are even more prohibitively priced, and in many regions not available for sale. Recognizing the limited affordability of nets, the Tanzanian government provides vouchers for subsidized nets to parents of children under five and pregnant women for TSH 2500 (US$2.80) [1,2,4,12]. However, even with this voucher programme, only 33.8% of children and 29% of pregnant women reported sleeping under nets in Kagera in 2007-2008 [4]. Consistent with these statistics, one critique of voucher programs is that they tend to favour the better-off. It is therefore not surprising that free distribution interventions were found to achieve the highest coverage among the poorest populations [13].

In the past, the most successful bed net implementation projects have employed some form of an educational model alongside the provision of nets. Often this model involved only simple instructions for net set-up and use. While several studies have shown that combined education and distribution results in significantly increased net usage, coverage rates still hover at 49 to 67.3% [13,14]. Other studies have shown maximum estimates of net coverage reaching 70.6% in the year of distribution, but falling to 62.8% and 35.4% in the following two years [15]. These rates are well below the estimated 80% coverage needed to achieve "mass effect," and are, therefore, suboptimal in reducing malaria transmission.

One factor contributing to low net coverage rates is the impact of local customs on net usage. For example, a study in Kenya found that the youngest children in a household were given the lowest priority for bed net use, despite being a higher-risk population [16]. A study in Burkina Faso showed decreased use of bed nets during the dry season due to a perceived lower risk of mosquito bites and the practice of sleeping outdoors [17]. A study in Kenya showed that villagers were using their nets for fishing rather than malaria prevention [18].

It is clear that the provision of LLINs and education are both instrumental for attaining high bed net coverage and usage rates. Yet, community-specific behaviours remain significant obstacles to achieving optimal results. Therefore, the objective of this project was to combine free LLIN distribution with a novel educational model targeting the unique beliefs and behaviours of bed net-receiving communities.

## Methods

A primary census of Ahakishaka village in the Kagera region was conducted, during which all counted households were assigned a number. Ten households were selected by systematic random sampling for interviews conducted in Swahili and/or Kinyambo, the local tribal language. Interviews were conducted in individual homes with various family members present. The research team encouraged participants to engage in open conversation while mediating to ensure that all salient issues were adequately addressed. Topics discussed during these interviews included: 1) malaria endemicity: experienced clinical symptoms, frequency of malaria-related clinic visits, and treatments; 2) malaria net use: current number of nets per household, net purchasing locations, correct use, maintenance, perceived efficacy in preventing malaria transmission, and reasons precluding net purchase or use; 3) economic barriers to net procurement; and 4) cultural beliefs that might deter use.

Based on interview responses, the research team identified five common local practices and attitudes inconsistent with effective bed net use: 1) bed net use is unnecessary during the dry season; 2) all nets need to be retreated after washing; 3) damaged nets cannot be repaired and should be discarded; 4) young children do not need to use nets during daytime and evening naps; and 5) bed nets are not a financial priority for families.

To confirm the prevalence of these five practices and attitudes, a five-question "Pre-Education" survey was developed. One hundred thirty-four households were selected by systematic random sampling to attend an official "Pre-Education" survey day. From each household, one female and one male representative 15 years or older were invited to participate. Population data including prior bed net ownership, family size, and the number of children in each household was collected from participants prior to survey administration. Surveys were composed in Swahili with choices of "yes," "no," or "I don't know" offered for each question. For residents who only spoke Kinyambo, a translator was utilized. Correct responses indicated: 1) bed net use is necessary during the dry season; 2) LLINs do not need to be retreated after washing; 3) damaged nets should be repaired; 4) young children should use nets during naps; and 5) bed nets are a financial priority. "I don't know" responses were always considered incorrect.

Following survey administration, a net "Distribution Day" was scheduled for each of the five subdivisions of Ahakishaka. "Distribution Days" included an LLIN "Education Session" presented by local leaders including the Village Chairman, Chief Medical Officer, the five subdivision leaders, and a local NGO coordinator. This session addressed those practices and attitudes found to be prevalent in "Pre-Education" survey responses, as well as the local impact of malaria infection, including incidence rates. In addition to a demonstration of LLIN maintenance and proper use, the session highlighted the differences between long-lasting nets and the retreatment-dependent nets available for purchase in Ahakishaka. Residents were advised of the need to supervise children napping under nets. Every household was encouraged to share responsibility in using distributed nets consistently and correctly. Following "Education Sessions", Olyset LLINs were distributed. Households with more than two adults, multiple children, or with one child over the age of five received two nets. Households with two or fewer adults and only one child under the age of five received one net.

During "Distribution Days", 264 participants representing 132 households were selected by systematic random sampling to attend a "Post-Education" survey three weeks later. This short-term follow-up survey addressed the same five practices and attitudes.

For the 15-month follow-up surveys, 256 participants representing 128 households were selected by systematic random sampling. These surveys evaluated the same five practices and attitudes as the "Pre-Education" and "Post-Education" surveys, with two additional questions regarding: 1) if the participants attended the education session, and 2) if the participants had used their net(s) everyday since distribution. Researchers also visited the selected households to confirm that Olyset LLINs were hanging over beds and in use.

Participants for each of the three surveys were selected by systematic random sampling, with every fourth household invited for survey. Not all of the selected household members were present on survey days; therefore, a total of 132 villagers participated in the "Pre-Education" survey, 104 in the "Post-Education" survey, and 104 in the 15-month follow-up survey. "Post-Education" and 15-month follow-up surveys were constructed using parallel forms in which questions were reworded to avoid a practice effect. "Post-Education" and 15-month follow-up survey responses were compared to "Pre-Education" survey responses. Changes in baseline attitudes and practices were analysed by intent-to-treat analysis, whereby all answers were included regardless of respondent attendance at the "Education Session." Significance was determined by chi-square test. A Bonferroni correction was applied, since one survey was used to address all five attitudes and practices, and a p-value of < 0.01 was accepted as significant.

"Pre-Education," education, distribution, and "Post-Education" stages of the project were carried out from June to August 2007, during the Kagera region's dry season. Fifteen-month follow-up data was collected in September 2008, also during the dry season. There were no significant changes in climate or population figures during these two periods.

## Results

Primary village census revealed 539 households with a population of 2,716 residents, according to the last regional census in 2002 [19]. Data collected on the day of the "Pre-Education" survey revealed no bed net ownership among the 132 participants. Population demographics showed an average family size of 5.86 people, with a ratio of 2.6 "children" to one adult. Children were defined as anyone under the age of 15. The surveyable population included all Ahakishaka residents who were 15 years and older, and was calculated as approximately 1,057 residents.

**Figure 1:**
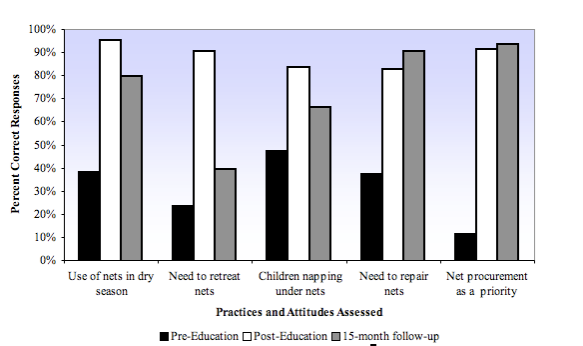
**Percent correct responses to surveys assessing community-specific practices and attitudes**. This graph depicts percent correct responses to survey questions assessing attitudes and practices regarding proper malaria net use. "Post-Education" and 15-month follow-up survey results show significant increases in "correct" responses from baseline.

"Pre-Education" surveys (n = 132) confirmed the prevalence of all five practices and attitudes identified during preliminary household interviews, with 53% to 88.6% incorrect responses (11.4%-47% correct responses) (Table 1; Figure 1). "Post-Education" surveys (n = 104) showed significant increases in correct responses in all five practices and attitudes from "Pre-Education" surveys (p < 0.01). Correct responses increased: 1) from 37.9% to 95.2% in "it is necessary to use nets in dry season"; 2) from 23.5% to 90.4% in "it is not necessary to retreat LLINs after washing"; 3) from 47.0% to 83.7% in "children should nap under nets"; 4) from 37.1% to 82.7% in "damaged nets should be repaired"; 5) from 11.4% to 91.3% in "net procurement is a financial priority" (Table 1; Figure 1).

**Table 1:** Change in survey responses from "Pre-Education" to "Post-Education" surveys

	"Pre-Education" (n = 132)			"Post-Education" (n = 104)			
	***Correct***	***Incorrect***	***% Correct***	***Correct***	***Incorrect***	***% Correct***	***p-value***

*Use of nets in dry season*	50	82	37.9%	99	5	95.2%	<0.001
*Need to retreat LLINs*	31	101	23.5%	94	10	90.4%	<0.001
*Children napping under nets*	62	70	47.0%	87	17	83.7%	<0.001
*Need to repair nets*	49	83	37.1%	86	18	82.7%	<0.001
*Net procurement as a priority*	15	117	11.4%	95	9	91.3%	<0.001

Fifteen-month follow-up surveys (n = 104) also showed significant increases in correct responses in all five practices and attitudes from "Pre-Education" surveys (p < 0.01). Correct responses increased: 1) from 37.9% to 79.8% in "it is necessary to use nets in dry season"; 2) from 23.5% to 39.4% in "it is not necessary to retreat LLINs after washing"; 3) from 47.0% to 66.3% in "children should nap under nets"; 4) from 37.1% to 90.4% in "damaged nets should be repaired"; 5) from 11.4% to 93.3% in "net procurement is a financial priority" (Table 2; Figure 1). 89.4% of participants reported using their LLINs everyday since distribution. This number was supported by direct inspection of nets hanging correctly during household visits.

**Table 2:** Change in survey responses from "Pre-Education" to 15-month follow-up surveys

	"Pre Education" (n = 132)			15-month follow-up (n = 104)			
	***Correct***	***Incorrect***	***% Correct***	***Correct***	***Incorrect***	***% Correct***	***p-value***

*Use of nets in dry season*	50	82	37.9%	83	21	79.8%	<0.001
*Need to retreat LLINs*	31	101	23.5%	41	63	39.4%	0.00829
*Children napping under nets*	62	70	47.0%	69	35	66.3%	<0.001
*Need to repair nets*	49	83	37.1%	94	10	90.4%	<0.001
*Net procurement as a priority*	15	117	11.4%	97	7	93.3%	<0.001

## Discussion

Household interviews both confirmed and rejected well-known practices and attitudes inconsistent with proper LLIN use, as gathered from a review of the literature and discussion with local NGOs. For example, male heads of household in Ahakishaka affirmed that young children have priority in sleeping under nets, in contrast to previously noted net use behaviours in East Africa. Other anecdotal practices reported, such as using nets for fishing and reticence to use nets during sexual intercourse were not validated by Ahakishaka residents and were, therefore, not addressed in "Pre-Education" surveys [18,20]. Only those issues pertinent to Ahakishaka with potential for modification were further investigated, including barriers to consistent net use that had not previously been described in the literature.

The five topics addressed in the "Pre-Education" survey were chosen based on their prevalence among interviewees and on their potential to preclude daily, effective net use. For example, interviewees reported that retreatment of nets presented an obstacle to use given both the cost and inconsistent availability of insecticide from local merchants. Nets remaining untreated were perceived as less effective in malaria prevention and were not used on a daily basis. Therefore, emphasizing the longevity of the insecticide in LLINs was key in promoting consistent use in this net-receiving community.

"Post-Education" surveys were conducted following a three-week interval in order to ensure that community-specific barriers were adequately addressed through the "Education Session." Results indicated that villagers improved most significantly in their consideration of net procurement as a priority, confirming the utility of the "Education Session" in modifying not only behaviours but also attitudes towards LLINs as a preventative health measure.

Fifteen-month follow-up assessed retention of knowledge and consistent use of nets in households. The number of correct responses to all questions remained significantly changed from baseline, but decreased from the "Post-Education" survey. The question assessing the need to retreat LLINs after washing had the lowest number of correct responses. This was likely due to confusion concerning the LLINs brought in by the research team versus the retreatment-dependent nets available locally. Decrease in correct responses may also be explained by the fact that, unlike the "Post-Education" survey in which all respondents attended the "Education Session," 15-month follow-up survey results were analysed via an intent-to-treat analysis including respondents who did not attend the session. The decrease in correct answers may also indicate the need for continued education after distribution. In the future, this could be accomplished through coordination with community health workers, volunteers and village leaders. Overall, 15-month follow-up validated the sustainability of the "Education Session" model, by showing continued statistically significant changes from baseline in all behaviours and attitudes.

Surveying the same individuals at baseline and follow-up was not feasible given the region's difficult terrain, time constraints, and the small size of the research team. Reliance on translators was also potentially problematic, as was the possibility for self-report bias when utilizing surveys to assess change. However, the use of questionnaires is well established in the literature of malaria bed net usage rates [21].

This study did not compare a concurrent non-educated control group with an educated intervention group. The main purpose of this intervention was to increase the correct usage of LLINs. To this end, the research team considered the provision of a net without education inconsistent with project goals. In addition, multiple studies have already established average net usage rates to be expected following interventions that combine net distribution with minimal or basic education [13-15]. Thus, the superior net usage rates achieved in this study 15 months following distribution validated the community-specific educational model.

A correlation between malaria incidence or prevalence and this intervention was beyond the scope of this project. Still, malaria incidence data was collected from the local health dispensary to compare the 12 month period before distribution from June 2006 to June 2007 with the 12 month period after distribution from June 2007 to June 2008. This data showed an incidence of 1,474 malaria cases for the 12 months before this intervention and an incidence of 1,068 malaria cases for the 12 months after this intervention. Several factors diminish the validity of this data. In Ahakishaka, malaria is diagnosed clinically, without the use of rapid testing, and is likely over-diagnosed, as in most endemic areas [22]. Also, the malaria incidence data obtained only reflects cases treated at the local health dispensary. Finally, though village leaders affirm that the population size was stable and there was no significant change in the number of visits to the dispensary, there was not a defined denominator; therefore, an absolute reduction in the incidence rate could not be determined. Nonetheless, the data may represent a trend toward decreased malaria incidence that should be more accurately assessed in future projects.

Community involvement and investment were essential to the design of this project. Preliminary meetings with village leaders sought to establish their interest in the proposed interventions, their goals and concerns, and suggestions on how to proceed. The research team clarified its role as facilitators in partnership with village volunteers who would help implement and sustain the goals of the intervention. The team lived within the community, and conducted assessments walking from house to house, allowing community members to familiarize themselves with both the researchers and the proposed intervention.

## Conclusions

The results from this study suggest that a novel educational model addressing locally prevalent misconceptions prior to LLIN distribution can successfully promote consistent and correct LLIN use and lead to high net coverage rates. Results also indicate that sustainability can be achieved by fostering positive attitudes towards bed net ownership and financial investment in LLINs. Future LLIN distributions in the Kagera region should adapt the paradigm established in this project, and should also address the growing demand for LLINs.

## Competing interests

The authors declare that they have no competing interests.

## Authors' contributions

MW & CN developed the project design under the guidance of NH and carried out the first year implementation of the project along with PB. MW & CN conducted the 15-month follow-up assessments and interviews. DH & PB implemented the expansion to a neighbouring village under the guidance of NH. MW, CN, DH & NH drafted the manuscript. All authors read and approved the final manuscript.
